# Sensitivity to Oxidative Stress in DJ-1-Deficient Dopamine Neurons: An ES- Derived Cell Model of Primary Parkinsonism

**DOI:** 10.1371/journal.pbio.0020327

**Published:** 2004-10-05

**Authors:** Cecile Martinat, Shoshana Shendelman, Alan Jonason, Thomas Leete, M. Flint Beal, Lichuan Yang, Thomas Floss, Asa Abeliovich

**Affiliations:** **1**Departments of Pathology and Neurology, Center for Neurobiology and Behavior, and Taub Institute, Columbia UniversityNew York, New YorkUnited States of America; **2**Department of Neurology and Neuroscience, Weill Medical College of Cornell UniversityNew York, New YorkUnited States of America; **3**Institute of Developmental Genetics, GSF-National Research Center for Environment and HealthNeuherbergGermany

## Abstract

The hallmark of Parkinson's disease (PD) is the selective loss of dopamine neurons in the ventral midbrain. Although the cause of neurodegeneration in PD is unknown, a Mendelian inheritance pattern is observed in rare cases, indicating a genetic factor. Furthermore, pathological analyses of PD substantia nigra have correlated cellular oxidative stress and altered proteasomal function with PD. Homozygous mutations in *DJ-1* were recently described in two families with autosomal recessive Parkinsonism, one of which is a large deletion that is likely to lead to loss of function. Here we show that embryonic stem cells deficient in DJ-1 display increased sensitivity to oxidative stress and proteasomal inhibition. The accumulation of reactive oxygen species in toxin-treated DJ-1-deficient cells initially appears normal, but these cells are unable to cope with the consequent damage that ultimately leads to apoptotic death. Furthermore, we find that dopamine neurons derived from in vitro–differentiated DJ-1-deficient embryonic stem cells display decreased survival and increased sensitivity to oxidative stress. These data are consistent with a protective role for DJ-1, and demonstrate the utility of genetically modified embryonic stem cell–derived neurons as cellular models of neuronal disorders.

## Abstract 2

Dopaminergic neurons, derived from DJ-1-deficient embryonic stem cells, display decreased survival and increased sensitivity to oxidative stress

## Introduction

Parkinson's disease (PD) is a progressive neurodegenerative disorder characterized by rigidity, slowed movement, gait difficulty, and tremor at rest (Dauer and Przedborski 2003). The pathological hallmark of PD is the relatively selective loss of dopamine neurons (DNs) in the substantia nigra pars compacta in the ventral midbrain. Although the cause of neurodegeneration in PD is unknown, a Mendelian inheritance pattern is observed in approximately 5% of patients, suggesting a genetic factor. Extremely rare cases of PD have been associated with the toxin 1-methyl-4-phenyl-1,2,3,6-tetrahydropyridine, which is taken up specifically by DNs through the dopamine transporter and is thought to induce cellular oxidative stress. Population-based epidemiological studies have further supported roles for genetic and environmental mechanisms in the etiology of PD (Dauer and Przedborski 2003; Jenner 2003).

The identification of several genes that underlie familial forms of PD has allowed for the molecular dissection of mechanisms of DN survival. Autosomal dominant mutations in *α-synuclein* lead to a rare familial form of PD (Polymeropoulos et al. 1997), and there is evidence that these mutations generate toxic, abnormal protein aggregates (Goldberg and Lansbury 2000) and cause proteasomal dysfunction (Rideout et al. 2001). A majority of patients with sporadic PD harbor prominent intracytoplasmic inclusions, termed Lewy bodies, enriched for α-synuclein (Spillantini et al. 1998), as well as neurofilament protein (Trojanowski and Lee 1998). Mutations in a second gene, *Parkin,* lead to autosomal recessive PD (Hattori et al. 2000). Parkin is a ubiquitin ligase that appears to participate in the proteasome-mediated degradation of several substrates (Staropoli et al. 2003).

Homozygous mutations in a third gene, *DJ-1,* were recently associated with autosomal recessive primary parkinsonism (Bonifati et al. 2003). *DJ-1* encodes a ThiJ domain protein of 189 amino acids that is broadly expressed in mammalian tissues (Nagakubo et al. 1997). Interestingly, DJ-1 was independently identified in a screen for human endothelial cell proteins that are modified with respect to isoelectric point in response to sublethal doses of paraquat (Mitsumoto and Nakagawa 2001; Mitsumoto et al. 2001), a toxin that generates reactive oxygen species (ROS) within cells and has been associated with DN toxicity (McCormack et al. 2002). Gene expression of a yeast homolog of DJ-1, YDR533C, is upregulated in response to sorbic acid (de Nobel et al. 2001), an inducer of cellular oxidative stress. These results suggest a causal role for DJ-1 in the cellular oxidative stress response.

Surprisingly, animal models that harbor genetic lesions that mimic inherited forms of human PD, such as homozygous deletions in *Parkin* (Goldberg et al. 2003; Itier et al. 2003) or overexpression of α-synuclein (Masliah et al. 2000; Giasson et al. 2002; Lee et al. 2002), have failed to recapitulate the loss of dopamine cells. An alternative approach, the genetic modification of midbrain DNs in vitro (Staropoli et al. 2003), is potentially useful but limited by the difficulty and variability in culturing primary postmitotic midbrain neurons. Other studies have focused on immortalized tumor cell lines, such as neuroblastoma cells, but these may not accurately model the survival of postmitotic midbrain neurons.

Here we show that DJ-1-deficient cells display increased sensitivity to oxidative stress. DNs appear to be particularly sensitive to the loss of DJ-1. The initial accumulation of ROS is normal in DJ-1-deficient cells, but subsequent cellular defenses to ROS are impaired, leading to increased apoptosis.

## Results

### Generation of DJ-1-Deficient ES Cells

**Figure 1:**
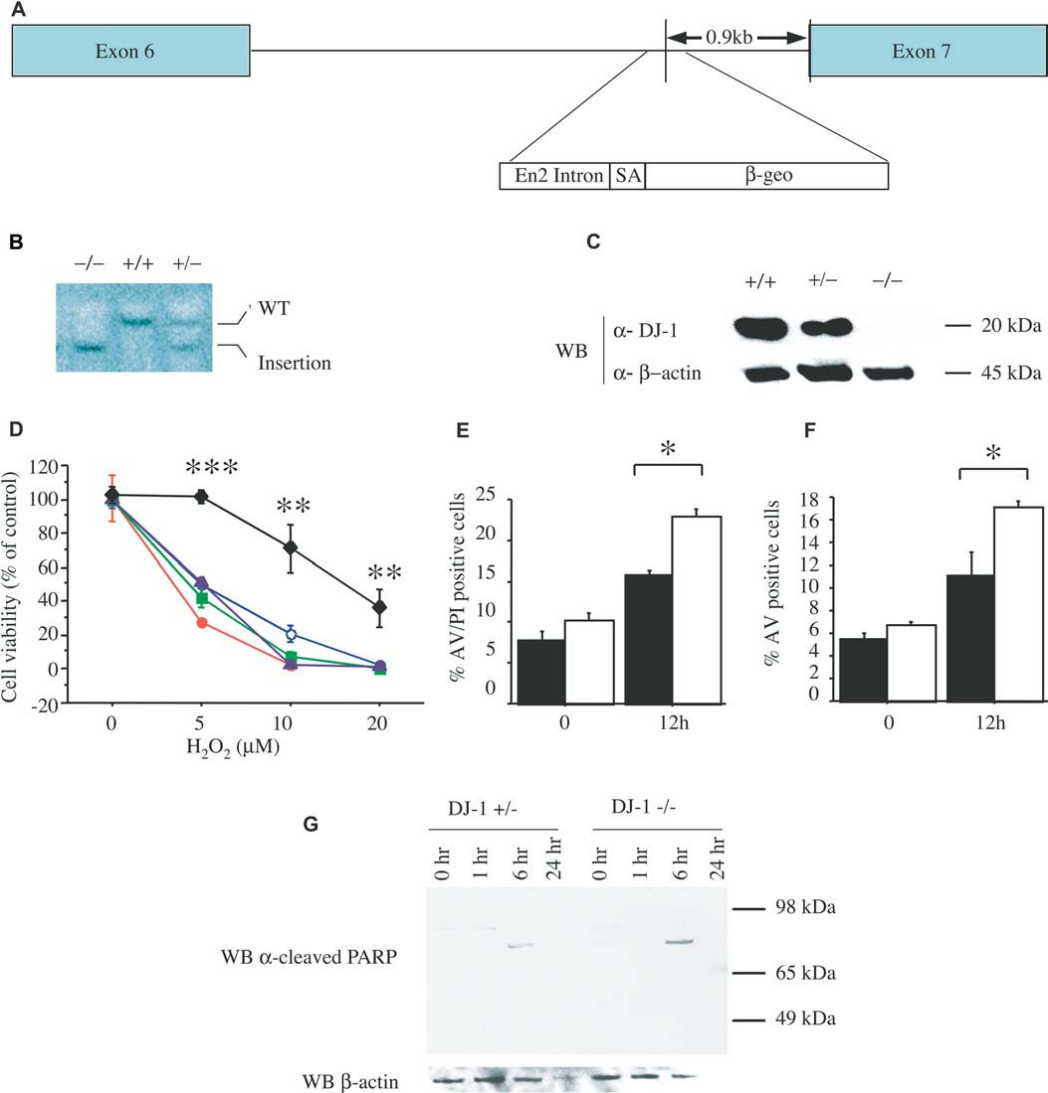
DJ-1-Deficient ES Cells Are Sensitized to Oxidative Stress (A) Schematic map of the murine *DJ-1* gene in clone F063A04. The retroviral insertion places the engrailed-2 (En2) intron, the splice acceptor (SA), and the β-galactosidase/neomycin resistance gene fusion (β-geo) between exons 6 and 7. (B) Southern blot analysis of KpnI-digested genomic DNA from *DJ-1* homozygous mutant (–/–), WT (+/+), and heterozygous (+/–)cells, probed with murine *DJ-1* cDNA. WT DNA shows a predicted 14-kb band (WT), whereas the mutant allele migrates as a 9-kb band (insertion). (C) Western blot (WB) of ES cell lysates from WT (+/+), *DJ-1* heterozygous (+/–), and mutant homozygous (–/–) clones with antibodies to murine DJ-1 (α-DJ-1) or β-actin (α-β-actin). DJ-1 migrates at 20 kDa, β-actin at 45 kDa. (D) ES cells were exposed to 0, 5, 10, and 20 μM H_2_O_2_ for 15 h and viability was assayed by MTT. Responses of *DJ-1* heterozygous cells (diamonds) and *DJ-1* knockout clones 9 (open circles), 16 (solid circles), 23 (squares), and 32 (triangles) are shown. ** *p* ≤ 0.01; *** *p* ≤ 0.0001. (E and F) Cell death of *DJ-1* heterozygous and DJ-1-deficient cells (clone 32) after exposure to H_2_O_2_ (10 μM) was quantified by staining with PI and an antibody to AV with subsequent FACS analysis. AV staining marks cells undergoing apoptosis, whereas PI staining indicates dead cells. * *p* ≤ 0.05. (G) *DJ-1* heterozygous (+/–) and knockout (clone 32; –/–) cells were assayed at 1, 6, and 24 h after treatment with 10 μM H_2_O_2_ by Western blotting for cleaved PARP (89 kDa), which indicates apoptosis. No band is seen for cleaved PARP or β-actin for the DJ-1-deficient cells at 24 h due to cell death. Data represent means ± standard error of the mean (SEM) and were analyzed by ANOVA with Fisher's post-hoc test.

To investigate the normal cellular function of DJ-1 and the pathogenic mechanism of the PD mutations, we generated cells deficient in DJ-1. A murine embryonic stem (ES) cell clone, F063A04, that harbors a retroviral integration at the *DJ-1* locus was obtained through the German Gene Trap Consortium (http://tikus.gsf.de) (Figure 1A; Floss and Wurst 2002). This integration is predicted to disrupt the normal splicing of *DJ-1,* leading to the generation of a truncated protein that lacks the carboxy-terminal domain required for dimerization and stability (unpublished data). Of note, a mutation that encodes a similarly truncated protein (at the human *DJ-1* exon 7 splice acceptor) has been described in a patient with early-onset PD (Hague et al. 2003).

To generate ES cell subclones homozygous for the trapped *DJ-1* allele, clone F063A04 was exposed to a high dose of the antibiotic G418, which selects cells that are homozygous for the neomycin resistance gene insertion (Mizushima et al. 2001). Several homozygous mutant ES cell subclones (that had undergone gene conversion at the *DJ-1* locus) were identified by Southern blotting (Figure 1B). To confirm that the trapped allele leads to the loss of wild-type (WT) DJ-1 protein, cell lysates from homozygous DJ-1-deficient (also termed “knockout” here) ES cell clones as well as the parental heterozygous clone were analyzed by Western blotting using polyclonal antibodies to the amino-terminal region of DJ-1 (amino acids 64–82) or full-length DJ-1 protein (unpublished data). Neither full-length nor truncated DJ-1 protein products were detected in knockout clones (Figure 1C), consistent with instability of the predicted truncated DJ-1 product. In addition, no full-length *DJ-1* RNA was detected in cultures of knockout cells (Figure S1). In contrast, heterozygous and WT ES cells express high levels of DJ-1. Initial phenotypic analysis of knockout subclones indicated that DJ-1 is not essential to the growth rate of ES cells in culture, consistent with the viability of humans homozygous for *DJ-1* mutations.

### DJ-1 Protects Cells from Oxidative Stress and Proteasomal Inhibition

**Figure 2:**
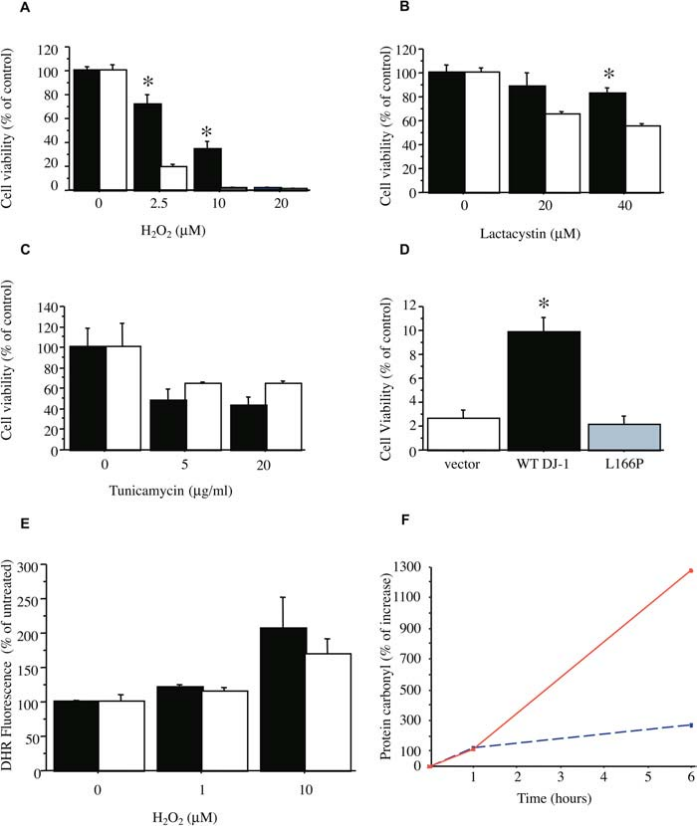
Specificity and Mechanism of Altered Toxin Sensitivity in DJ-1-Deficient Cells (A–C) Cell viability of *DJ-1* heterozygous cells (solid bar) and DJ-1-deficient knockout clone 32 cells (open bar) after 15 h exposure to H_2_O_2_ (A), lactacystin (B), or tunicamycin (C) as assayed by MTT reduction. * *p* ≤ 0.05. (D) DJ-1-deficient knockout cells (clone 32) were transiently transfected with plasmids containing WT human DJ-1 vector (solid bar) and PD-associated L166P mutant DJ-1 vector (gray bar); as a control, knockout cells were also transfected with vector alone (open bar). 48 h after transfection, cells were exposed to 10 μM H_2_O_2_ for 15 h and then assayed by MTT reduction. WT human DJ-1 significantly enhanced survival of the knockout cells, whereas the L166P mutant did not. Similar results were obtained at 20 μM H_2_O_2_ and with a second DJ-1-deficient clone (unpublished data). Transfection efficiency exceeded 90% in all cases and protein expression level was comparable for human WT and L166P mutant DJ-1 as determined by Western blotting (Figure S1). * *p* ≤ 0.05. (E) DJ-1-deficient cells (clone 32; open bar) and control heterozygous cells (solid bar) were assayed for intracellular formation of ROS in response to H_2_O_2_ treatment (15 min, 1 or 10 μM) using DHR and FACS analysis. (F) Protein carbonyl levels were measured by spectrophotometric analysis of DNP-conjugated lysates from DJ-1-deficient (clone 32; solid red line) and control heterozygous cells (dashed blue line). Data are shown as the mean ± SEM and were analyzed by ANOVA with Fisher's post-hoc test.

DJ-1 has been hypothesized to function in the cellular response to oxidative stress. To investigate the role of DJ-1 in the oxidative stress response in vivo, DJ-1-deficient knockout and heterozygous ES cell clones were analyzed for cell viability in the context of increasing concentrations of H_2_O_2_. Heterozygous cells were used as controls because the knockout subclones were derived from these. Cell viability was initially determined by MTT assay (which detects reduction of 3-(4,5-dimethylthiazol-2-yl)-2,5-diphenyltetrazolium bromide [MTT] by metabolic enzymes) in triplicate (Fezoui et al. 2000). Exposure to H_2_O_2_ led to significantly greater toxicity in the DJ-1-deficient cells; similar results were obtained with multiple knockout subclones in independent experiments (Figures 1D and 2A). In contrast, in the absence of toxin, heterozygous and knockout cells displayed comparable viability in the MTT assay (Figure S2). Consistent with the MTT assay, fluorescence-activated cell sorting (FACS) analysis of cells stained with annexin V (AV) and propidium iodide (PI) revealed increased death of knockout cells compared to heterozygous cells in the context of H_2_O_2_ exposure (Figure 1E). The increase in AV-positive cells implicated an apoptotic mechanism of cell death (Figure 1F). Furthermore, when exposed to H_2_O_2_, knockout cells displayed potentiated cleavage of poly(ADP-ribose)polymerase-1 (PARP) in a pattern indicative of an apoptotic death program (Gobeil et al. 2001) (Figure 1G).

Additional toxin exposure studies demonstrated that DJ-1-deficient cells were sensitized to the proteasomal inhibitor lactacystin (Figure 2B), as well as to copper (Figure S2), which catalyzes the production of ROS. We did not observe altered sensitivity to several other toxins, including tunicamycin (an inducer of the unfolded protein response in the endoplasmic reticulum; Figure 2C), staurosporine (a general kinase inhibitor that induces apoptosis) (Figure S2), or cycloheximide (an inhibitor of protein translation) (unpublished data).

### WT but Not PD-Associated L166P Mutant DJ-1 Protects Cells from Oxidative Stress

To confirm that altered sensitivity to oxidative stress is a consequence of the loss of DJ-1, we performed rescue experiments by overexpressing WT or mutant human DJ-1 in knockout ES cells. Plasmids encoding human Flag-tagged WT DJ-1, Flag-tagged PD-associated L166P mutant DJ-1, or vector alone, were transiently transfected into DJ-1-deficient clones, and these were subsequently assayed for sensitivity to H_2_O_2_ using the MTT viability assay. DJ-1-deficient cells transfected with a vector encoding Flag-WT human DJ-1 were effectively rescued in terms of viability in the presence of H_2_O_2_ (Figure 2D); Thus, viability in rescued knockout cells mimicked the viability of untransfected heterozygous cells in the context of H_2_O_2_ treatment (Figure 2A and 2D). In contrast, transfection of knockout cells with a vector encoding the PD-associated L166P mutant DJ-1 did not significantly increase the viability of H_2_O_2_-treated knockout cells (Figure 2D). Baseline cell viability in the absence of toxin exposure was not altered by DJ-1 overexpression, and Western blotting of lysates from transfected cells with an antibody specific to human DJ-1 demonstrated that transfected Flag-WT DJ-1 and Flag-L166P mutant DJ-1 accumulated comparably (Figure S2).

### DJ-1 Deficiency Does Not Alter the H_2_O_2_-Induced Intracellular ROS Burst

We hypothesized that DJ-1 either alters the initial accumulation of intracellular ROS in response to H_2_O_2_ exposure, or that it functions downstream of the ROS burst and protects cells from consequent damage. Therefore, we quantified the accumulation of ROS in response to H_2_O_2_ treatment in knockout and heterozygous cells using the ROS-sensitive fluorescent indicator dye dihydrorhodamine-123 (DHR) and FACS analysis. Initial ROS accumulation (at 15 min after stimulation) appeared unaltered in the DJ-1-deficient cells in comparison to control heterozygous cells (Figure 2E). Consistent with this, accumulation of protein carbonyls, an index of oxidative damage to proteins (Sherer et al. 2002), appeared normal initially (at 1 h after toxin exposure; Figure 2F). However, at 6 h after toxin exposure, a point at which knockout cells already display increased apoptosis (as indicated by PARP cleavage; see Figure 1G), protein carbonyl accumulation was robustly increased in the DJ-1-deficient cells. These data suggest that initial ROS accumulation is not altered by DJ-1 deficiency, but that the mutant cells are unable to appropriately cope with the consequent damage. Consistent with this result, no antioxidant or peroxiredoxin activity with purified DJ-1 protein in vitro was detected (S.S. and A.A., personal communication).

### DJ-1 Is Required for Survival of ES Cell–Derived DNs

**Figure 3:**
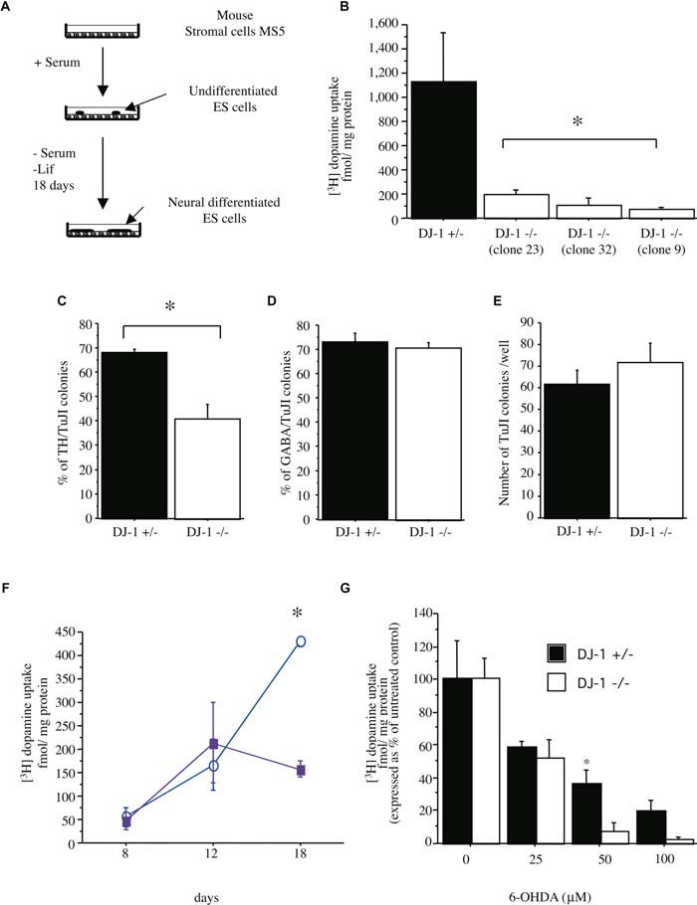
DJ-1-Deficient ES Cell Cultures Display Reduced DN Production (A) The SDIA coculture method. *DJ-1* knockout or control heterozygous ES cells are cocultured with mouse stromal cells (MS5) in the absence of serum and leukemia inhibitory factor for 18 DIV. (B) DN production was quantified at 18 DIV by ^3^H-dopamine uptake assay. DJ-1-deficient ES cell cultures were defective relative to heterozygous control cultures. (C–D) Neuron production was quantified by immunohistochemical analysis as a percent of TuJ1-positive colonies that express TH (C) or GABA (D). Quantification of TH and GABA immunostaining was performed on all colonies in each of three independent wells. Colonies were scored as positive if any immunostained cells were present. * *p* ≤ 0.05. (E) The absolute number of TuJ1-positive colonies was not significantly different between the two genotypes. (F) Kinetic analysis of DN differentiation in DJ-1-deficient cultures (clone 32, solid square) and heterozygous controls (open circle) as quantified by ^3^H-dopamine uptake assay. * *p* ≤ 0.05. (G) DJ-1-deficient (open bar) and heterozygous control (closed bar) cultures differentiated for 9 DIV and then exposed to 6-OHDA at the indicated dose for 72 h. DNs were quantified by ^3^H-dopamine uptake assay. Data represent the means ± SEM and were analyzed by ANOVA followed by Fisher's post-hoc test. * *p* ≤ 0.05.

**Figure 4:**
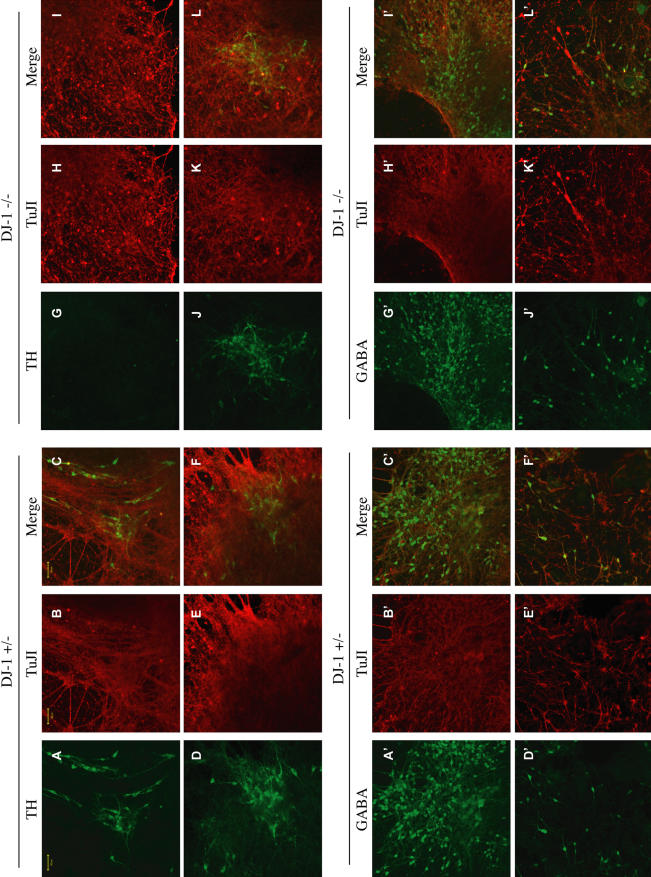
Neuronal Differentiation of DJ-1-Deficient and Control Heterozygous ES Cell Cultures (A–L) *DJ-1* heterozygous (+/–; A–F) and knockout (–/– [clone 32]; G–L) cultures were differentiated by SDIA for 18 DIV and immunostained with antibodies to TH (green) and TuJ1 (red). Images of both (Merge) are also shown. (A′–L′) Immunostaining of *DJ-1* heterozygous (+/–, A′–F′) and deficient (–/–, G′–L′) cultures with antibodies for GABA (green) and TuJ1 (red). Scale bar, 50 μm. Images of both (Merge) are also shown.

Several methods have been established for the differentiation of ES cells into DNs in vitro (Morizane et al. 2002). To extend our analysis of DJ-1 function to DNs, we differentiated DJ-1-deficient ES cells or control heterozygous cells into DNs in vitro by coculture with stromal cell–derived inducing activity (SDIA; Figure 3A) (Morizane et al. 2002; Barberi et al. 2003). DNs were quantified by immunohistochemistry for tyrosine hydroxylase (TH; a marker for DNs and other catecholaminergic cells), or by analysis of dopamine transporter uptake activity (a quantitative DN marker) (Han et al. 2003). Production of DNs appeared to be significantly reduced in knockout ES cell cultures compared to parental heterozygous cultures at 18 days in vitro (DIV) as determined by both dopamine uptake and TH immunoreactivity (Figures 3B and 3C; 4A–4L). In contrast, general neuronal production did not appear altered in this assay in terms of the postmitotic neuronal marker TuJ1 (a monoclonal antibody specific to neuronal, not glial, class III β-tubulin) (Figures 3E and 4A–4L′); other neuronal subtypes also appeared normal, including GABAergic (Figures 3D and 4A′–4L′) and motor neurons (HB9-positive; Figure S3). To investigate whether the reduction in DNs in DJ-1-deficient cultures is due to defective generation or survival, a time course analysis was performed. At early time points (8 and 12 DIV), dopamine uptake activity was comparable in WT and DJ-1-deficient cultures, whereas subsequently the DJ-1-deficient cultures appeared defective (Figure 3F). Consistent with this, intracellular dopamine accumulation (as quantified using high-performance liquid chromatography) was significantly reduced in DJ-1-deficient cultures (6.4 ± 1.5 ng dopamine/mg protein) relative to control heterozygous cultures (66.0 ± 17.4 ng/mg) at 35 DIV. These data strongly suggest that DJ-1 deficiency leads to loss of DNs, rather than simply to downregulation of cell marker expression.

We hypothesized that DJ-1-deficient DNs may be sensitized to oxidative stress, akin to DJ-1-deficient undifferentiated ES cells. To test this, DN cultures from DJ-1-deficient or heterozygous control ES cell cultures at 9 DIV were exposed to oxidative stress in the form of 6-hydroxydopamine (6-OHDA), a DN-specific toxin that enters DNs through the dopamine transporter and leads to oxidative stress and apoptotic death (Dauer and Przedborski 2003). DJ-1-deficient DNs displayed an increased sensitivity to oxidative stress in this assay (Figure 3G). Post-hoc analysis of the data indicates that the difference among genotypes is maximal at an intermediate dose of toxin (50 μM); at the highest dose of 6-OHDA employed (100 μM), the difference is lessened (because the heterozygote is increasingly affected as well), indicating that DJ-1-mediated protection is limited. Although we cannot exclude a role for DJ-1 in the late-stage differentiation of DNs, these data suggest that DJ-1 deficiency leads to reduced DN survival and predisposes these cells to endogenous and exogenous toxic insults.

### RNAi “Knockdown” of DJ-1 in Midbrain Embryonic DNs Leads to Increased Sensitivity to Oxidative Stress

**Figure 5:**
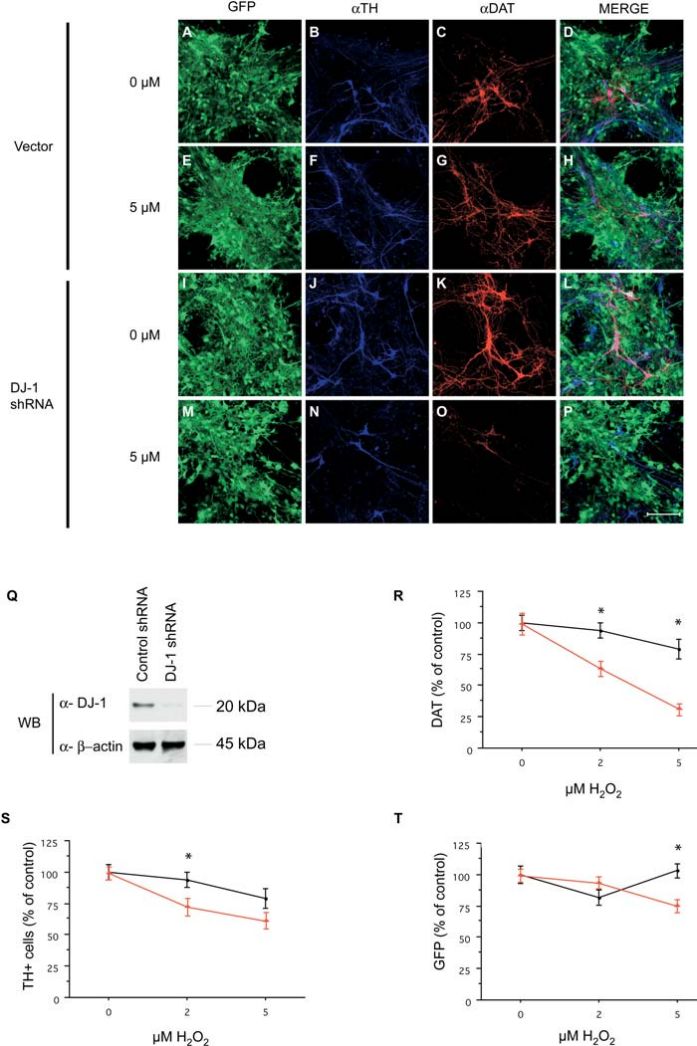
RNAi “Knockdown” of *DJ-1* in Primary Embryonic Midbrain DNs Display Increased Sensitivity to Oxidative Stress (A–P) Primary midbrain cultures from E13.5 embryos were infected with lentiviral vectors encoding *DJ-1* shRNA (or vector alone) under the regulation of the control vector (A–H) or the U6 promoter (I–P). Cells were cultured for 1 wk after infection and then exposed to H_2_O_2_ (5 μM; E–H and M–P) for 24 h. Cultures were immunostained for TH (B, F, J, and N) or DAT (C, G, K, or O) and visualized by confocal microscopy. Images containing all stains are included (Merge; D, H, L, and P). Scale bar, 100 μm. (Q) Cell lysates prepared from midbrain primary cultures infected with *DJ-1* shRNA lentivirus (or control vector) were analyzed by Western blotting for murine DJ-1 or β-actin. (R–T) Quantification of TH, DAT, and GFP signal was performed on ten randomly selected fields in each of three wells for each condition. Red triangles, *DJ-1* shRNA treated; black circles, control vector. Data represent the means ± SEM and were analyzed by ANOVA followed by Fisher's post-hoc test. * *p* ≤ 0.05.

To confirm the role of DJ-1 in primary midbrain DNs, DJ-1 expression was inhibited by RNA interference (RNAi) in embryonic day 13.5 (E13.5) murine primary midbrain cultures by lentiviral transduction of short hairpin RNAs (shRNAs) (Figure 5) (Rubinson et al. 2003). E13.5 midbrain cultures (Staropoli et al. 2003) were transduced with a lentiviral vector that includes a gene encoding the green fluorescent protein marker eGFP, along with shRNAs homologous to murine *DJ-1. DJ1*-shRNA virus-infected cells displayed efficient silencing of *DJ-1* gene expression to 10%–20% of control vector-infected cultures (as determined by Western blotting [Figure 5Q]). Transduction efficiency, as assessed by visualization of the fluorescent eGFP marker, exceeded 95% in all cases (Figure 5I and unpublished data). After 7 DIV, cultures were exposed to H_2_O_2_ for 24 h and then evaluated for DN survival as quantified by immunostaining for TH and dopamine transporter (DAT).

Midbrain cultures transduced with *DJ-1* shRNA virus and with control vector displayed similar numbers of TH-positive neurons in the absence of exposure to H_2_O_2_ (Figure 5A–D, 5I–L, and 5R–S). In contrast, in the presence of H_2_O_2_, DJ-1-deficient cultures displayed significantly reduced DN survival as quantified by immunohistochemistry for TH or DAT (Figure 5E–H, 5M–P, 5R–S). These studies were repeated three times with similar results. The reduction in DAT immunoreactivity appears to be more robust than the reduction in TH-positive cell number in the context of H_2_O_2_; this may reflect the differential localization of DAT to DN processes, whereas TH is primarily in the cell body. As we described in a previous manuscript, nondopaminergic cells in the E13.5 primary midbrain cultures are predominantly GABAergic neurons (90%–95%) (Staropoli et al. 2003). Total embryonic midbrain neurons transduced with either *DJ-1* shRNA or vector displayed comparable survival in the context of toxin exposure, suggesting that DJ-1 deficiency leads to a relatively specific alteration in DN survival (Figure 5T). These data are consistent with the analyses of ES cell–derived DNs above and indicate that DJ-1 is required for the normal survival of midbrain DNs in the context of toxin exposure.

## Discussion

In this study we present evidence that DJ-1 is an essential component of the oxidative stress response of DNs. DJ-1-deficient cells display an apparently normal initial burst of ROS in response to H_2_O_2_, but they are unable to cope with the consequent toxicity, culminating in apoptosis. Additionally, we find that DJ-1 deficiency sensitizes cells to the proteasomal inhibitor lactacystin but not other toxic stimuli such as tunicamycin. Proteasomal inhibition induces the accumulation of short-lived and misfolded cytoplasmic proteins, leading to oxidative stress and apoptosis (Demasi and Davies 2003). ROS and proteasomal inhibition have previously been correlated with PD pathology (Dauer and Przedborski 2003), and it is therefore tempting to hypothesize that *DJ-1* mutations lead to PD because of an increased sensitivity to such stressors.

The apparent cell-type specificity of DN impairment in patients with the Parkinsonism-associated *DJ-1* mutation is not predicted by the ubiquitous expression of DJ-1 (Nagakubo et al. 1997). In this study, we find that DJ-1 protects both dopaminergic and nondopaminergic cells from oxidative insult. However, DJ-1-deficient DNs appear to be especially sensitive to oxidative insult, suggesting relative cell-type specificity to the consequences of DJ-1 deficiency. Similar results are observed in DJ-1-knockout ES cell–derived DNs (which are devoid of any detectable DJ-1) and in primary DNs with DJ-1 levels reduced by RNAi “knockdown.” However, we find that even in the absence of exogenous toxin exposure, the knockout ES cell–derived DNs display reduced survival, whereas survival of the primary embryonic midbrain RNAi knockdown DNs appears to be similar to WT cells. We hypothesize that this discrepancy reflects the activity of residual DJ-1 (approximately 10%–20%) in the RNAi knockdown cultures. Alternatively, the knockout ES cell–derived DNs may be exposed to a greater degree of oxidative stress in vitro than are the knockdown-derived DNs even in the absence of added toxin. The mechanism by which DNs are preferentially targeted for destruction in the absence of DJ-1 is unclear. It has been proposed that DNs are subject to high levels of endogenous oxidative stress that may relate to dopamine metabolism (Jenner and Olanow 1998).

DJ-1 is structurally modified in the context of cellular oxidative stress (Mitsumoto and Nakagawa 2001), suggesting a possible function. Two recent studies (Yokota et al. 2003; Taira et al. 2004) investigated the role of DJ-1 in the oxidative stress response of neuroblastoma tumor cells. Both studies used RNAi to perturb the expression of DJ-1 in neuroblastoma tumor cell lines, and suggested that DJ-1 deficiency sensitizes cells to oxidative stress; these results are consistent with our data. Taira et al. (2004) further reported that overexpression of DJ-1 in neuroblastoma cells leads to a reduction in ROS accumulation and hypothesized that DJ-1 may harbor antioxidant activity in vivo. In contrast, we find that ES cells that are deficient in DJ-1 display a normal initial burst of ROS in the context of H_2_O_2_. Consistent with this, we fail to detect DJ-1 antioxidant activity in vitro (Shendelman et al. 2004).

Finally, this study presents a novel, ES cell-based genetic approach to the study of neurodegenerative disorders. Mouse genetic models of disease are often limited by the inherent variability of animal experiments, the limited mouse life span, and the difficulties in manipulating whole animals. For instance, genetic rescue experiments and toxicological dose-response studies are impractical in whole animals. Furthermore, genetic cell models are more readily amenable to molecular dissection of disease mechanisms than are whole animals. Thus, genetically altered, ES cell–derived neurons are likely to be generally useful as cellular models of neurodegenerative disorders. Future studies may also utilize available human ES cells to investigate species differences.

## Materials and Methods

### 

#### Cell culture.

Undifferentiated ES cells were cultured using standard techniques (Abeliovich et al. 2000). SDIA differentiation of ES cell cultures to DNs was performed as described in Kawasaki et al. (2000), except that ES cells were plated at a density of 500 cells/cm^2^ rather than approximatively 1,000 cells/cm^2^, and were cocultured with the MS5 mouse stromal cell line (Barberi et al. 2003). For rescue experiments, cells were plated at a cell density of 1.4 × 10^6^ cells/well. Transfections with plasmids encoding human Flag-WT DJ-1, PD-associated L166P mutant DJ-1, or vector alone, were performed using Lipofectamine 2000 (Life Technologies) for 18–36 h according to the manufacturer's instructions (Staropoli et al. 2003). 24 hours post-transfection, cells were split into 96-well plates and treated as described below. Primary cultures and lentiviral transductions were performed as described in Staropoli et al. (2003).

#### Generation of knockout ES cell clones

The pT1ATGβgeo gene trap vector, which includes βgeo, a fusion of the genes for β-galactosidase and neomycin resistance, is present between exons 6 and 7 of the murine *DJ-1* gene, as determined by cDNA sequencing of trapped transcripts and genomic analysis (Figure 1A). To select for ES cell subclones homozygous for the trapped *DJ-1* allele, we treated clone F063A04 with 4 mg/ml G418. Several subclones that were homozygous for the mutant *DJ-1* allele were identified by Southern blotting (see Figure 1B), and three were chosen for further experimentation: clones 9, 23, and 32. To confirm that the trapped allele leads to the loss of wild-type (WT) DJ-1 protein, cell lysates from these clones, as well as from the parental heterozygous clone, were analyzed by Western blotting using polyclonal antibodies to the amino-terminal region of DJ-1 or the full-length DJ-1 protein. For Western blotting, cells were resuspended in 50 mM Tris-HCl (pH 7.4), 150 mM NaCl, and 0.2% Triton X-100, and incubated at 4 °C, rotating for 20 min. Cleared lysate was prepared by centrifuging the lysate at 13,000 rpm for 10 min at 4 °C.

#### Antibodies.

A rabbit polyclonal antibody to DJ-1 was generated against the synthetic polypeptide QNLSESPMVKEILKEQESR, which corresponds to amino acids 64–82 of the mouse DJ-1 protein. Antiserum was produced by the Polyquick polyclonal antibody production service of Zymed Laboratories (South San Francisco, California, United States). The antiserum was affinity-purified on a peptide-coupled Sulfolink column (Pierce Biotechnology, Rockford, Illinois, United States) according to the manufacturer's instructions. Antibody was used at a dilution of 1:200 for immunohistochemistry and Western blotting as described (Staropoli et al. 2003). Immunohistochemistry was performed with a rabbit polyclonal antibody to TH (PelFreez, Rogers, Arizona, United States; dilution 1:1000), the mouse monoclonal antibody to neuronal class III β-tubulin TuJ1 (Covance, Princeton, New Jersey, United States; dilution 1:500), and a rabbit polyclonal antibody to GABA (Sigma, St. Louis, Missouri, United States; dilution 1:1000). Western blotting was performed using a polyclonal antibody to cleaved PARP (Cell Signaling Technology, Beverly, Massachusetts, United States; dilution 1:500), a monoclonal antibody to DJ-1 (Stressgen Biotechnologies, San Diego, California, United States; dilution 1:1000), and a mouse monoclonal antibody to β-actin (Sigma, 1:500).

#### In vivo assays.

ES cells plated in 96-well format (2.3 × 10^4^ cells/well) were treated for 15 h with H_2_O_2_ in ES cell medium deficient in β-mercaptoethanol (Abeliovich et al. 2000). Cell viability (as a percent of untreated control) was determined by MTT assay in triplicate (Fezoui et al. 2000). AV/PI (Molecular Probes, Eugene, Oregon, United States) staining was performed according to the manufacturer's instructions. For DHR staining (Molecular Probes) (Walrand et al. 2003), cells were preincubated for 30 min with DHR (5 μM), washed with PBS, then treated with H_2_O_2_ in ES cell medium deficient in β-mercaptoethanol for 15 min at 37 °C. The FACS analysis was performed using a FACSTAR sorter (Becton-Dickinson, Palo Alto, California, United States). Dopamine uptake assays were performed as described (Farrer et al. 1998). Reported values represent specific uptake from which nonspecific uptake, determined in the presence of mazindol, was subtracted. Uptake values were normalized for protein content with the BCA kit (Pierce).

For 6- hydroxydopamine (6-OHDA, Sigma) treatment, the drug was diluted in the differentiation medium (Kawasaki et al. 2000) and medium was changed every day for 72 h.

Primary midbrain embryonic cultures were prepared and transduced with lentiviral vectors as described in Staropoli et al. (2003). The *DJ-1* shRNA vector was generated by insertion of annealed oligonucleotides 5′-TGTCACTGTTGCAGGCTTGGTTCAAGAGACCAAGCCTGCAACAGTGACTTTTTTC-3′ and 5′-ACAGTGACAACGTCCGAACCAAGTTCTCTGGTTCGGACGTTGTCACTGAAAAAAGAGCT-3′ into the LentiLox vector (Rubinson et al. 2003). For cellular dopamine quantification, cultures were incubated in standard differentiation medium supplemented with L-DOPA (50 μM) for 1 h to amplify dopamine production, as described in Pothos et al. (1996). Subsequently, cells were washed in PBS and then lysed in 0.2 M perchloric acid. Dopamine levels were quantified by HPLC (Yang et al. 1998) and normalized for protein content as above.

#### Expression vectors.

The cDNA for human *DJ-1* was PCR-amplified from a human liver cDNA library (Clontech, Palo Alto, California, United States). For expression of DJ-1 in ES cell rescue experiments, *DJ-1* was cloned into the expression vector pcDNA3.1 (Invitrogen, Carlsbad, California, United States) containing a Flag peptide sequence at the N-terminus using standard cloning techniques. Flag-L166P *DJ-1* (pcDNA3) was generated by PCR-mediated mutagenesis.

#### Protein carbonyl analysis.

For protein carbonyl quantitation (Bian et al. 2003), cells were plated (1.4 × 10^5^ cells per well), grown for 24 h, and then treated with 10 μM H_2_O_2_ as indicated. Cells were lysed in 200 μl lysis buffer and cleared lysate was prepared as described above. An aliquot of 40 μl from each time point was added to 2 M HCl (120 μl) with or without 10 μM 2,4-dinitrophenyl-hydrazine and incubated for 1 h at 24 °C with shaking. Proteins were then TCA-precipitated and resuspended in 200 μl of 6 M guanidinium chloride. Absorbance was measured at 360 nm, and DNP-conjugated samples were normalized for protein concentration with the underivitized control samples.

## Supporting Information

Figure S1 Quantitative Real-Time PCR for *DJ-1* Gene Expression (A) Real-time PCR analyses of *DJ-1* cDNA in WT (+/+), heterozygous (+/–), and knockout (–/–) cultures. Each expression value was normalized to that of β-actin and expressed relative to the respective value of the WT (+/+) control. These gene expression patterns were replicated in at least three independent PCR experiments. Total RNA from ES cells differentiated with the SDIA method for 18 days was isolated using the Absolutely RNA Miniprep kit (Stratagene, La Jolla, California, United States). Synthesis of cDNA was performed using the SuperScript first strand synthesis system for RT-PCR (Invitrogen). Real-time PCR reactions were optimized to determine the linear amplification range. Quantitative real-time RT-PCRs were performed (Stratagene MX3000P) using the QuantiTect SYBR Green PCR Master Mix (Qiagen, Valencia, California, United States) according to the manufacturer's instructions. *DJ-1* primer sequences were 5′-CGAAGAAATTCGATGGCTTCCAAAAGAGCTCTGGT-3′ and 5′-CAGACTCGAGCTGCTTCACATACTACTGCTGAGGT-3′; primers used for β-actin were 5′-TTTTGGATGCAAGGTCACAA-3′ and 5′-CTCCACAATGGCTAGTGCAA-3′. For quantitative analyses, PCR product levels were measured in real time during the annealing step, and values were normalized to those of β-actin.
(B) Ethidium bromide staining of the PCR products obtained after 29 cycles for *DJ-1* (625 bp) and β*-actin* (350 bp).
(704 KB EPS).

Figure S2 Analysis of DJ-1-Deficient ES Cells (A and B) Cell viability of *DJ-1* heterozygous cells (solid bar) and DJ-1-deficient knockout clone 32 (open bar) after exposure to CuCl_2_ or staurosporine at the doses indicated.
(C) MTT values of untreated DJ-1-deficient ES cell clones and the control heterozygous cells. Assays were performed exactly as in Figure 2, but in the absence of toxin.
(D) MTT values of untreated DJ-1-deficient ES cells transfected with vector alone or various DJ-1-encoding plasmids. Transfection and expression of WT DJ-1 or mutant forms of DJ-1 does not alter the basal metabolic activity or viability of the cells.
(E) Western blotting of extracts from ES cells transfected with vectors harboring WT human *DJ-1* or the L166P mutant.
(545 KB EPS).

Figure S3 Immunocytochemistry for HB9 and GABA Neurons in DJ-1-Deficient and Control Heterozygous ES Cells Both cell cultures were differentiated by SDIA for 18 DIV. Cells were fixed with 4% paraformaldehyde and stained with mouse monoclonal antibodies against HB9 (gift from T. Jessell, dilution 1:50) and rabbit polyclonal antibodies against GABA (Sigma, dilution 1:1000) as in Figure 5. Scale bar, 50 μM.
(5.5 MB TIF).

### Accession Numbers

The GenBank (http://www.ncbi.nlm.nih.gov/) accession numbers of the genes discussed in this paper are *α-synuclein* (NM_000345), *Parkin* (AB009973), and *DJ-1* (AB073864).

## Abbreviations

6-OHDA: 6-hydroxydopamine
DAT: dopamine transporter
DHR: dihydrorhodamine-123
DIV: d in vitro
DN: dopamine neuron
E[number]: embryonic day [number]
ES: embryonic stem
FACS: fluorescence-activated cell sorter
MTT: 3-(4,5-dimethylthiazol-2-yl)-2,5-diphenyltetrazolium bromide
PARP: poly(ADP-ribose)polymerase-1
PI: propidium iodide
PD: Parkinson's disease
RNAi: RNA interference
ROS: reactive oxygen species
SDIA: stromal cell–derived inducing activity
SEM: standard error of the mean
shRNA: short hairpin RNA
TH: tyrosine hydroxylase
WT: wild-type
