# Supplementary figures and images for: Sensitivity to Oxidative Stress in DJ-1-Deficient Dopamine Neurons: An ES- Derived Cell Model of Primary Parkinsonism
Source: PLoS Biol. 2004 Oct 5;2(11):e327. doi: 10.1371/journal.pbio.0020327 (PMC521171; doi:10.1371/journal.pbio.0020327)

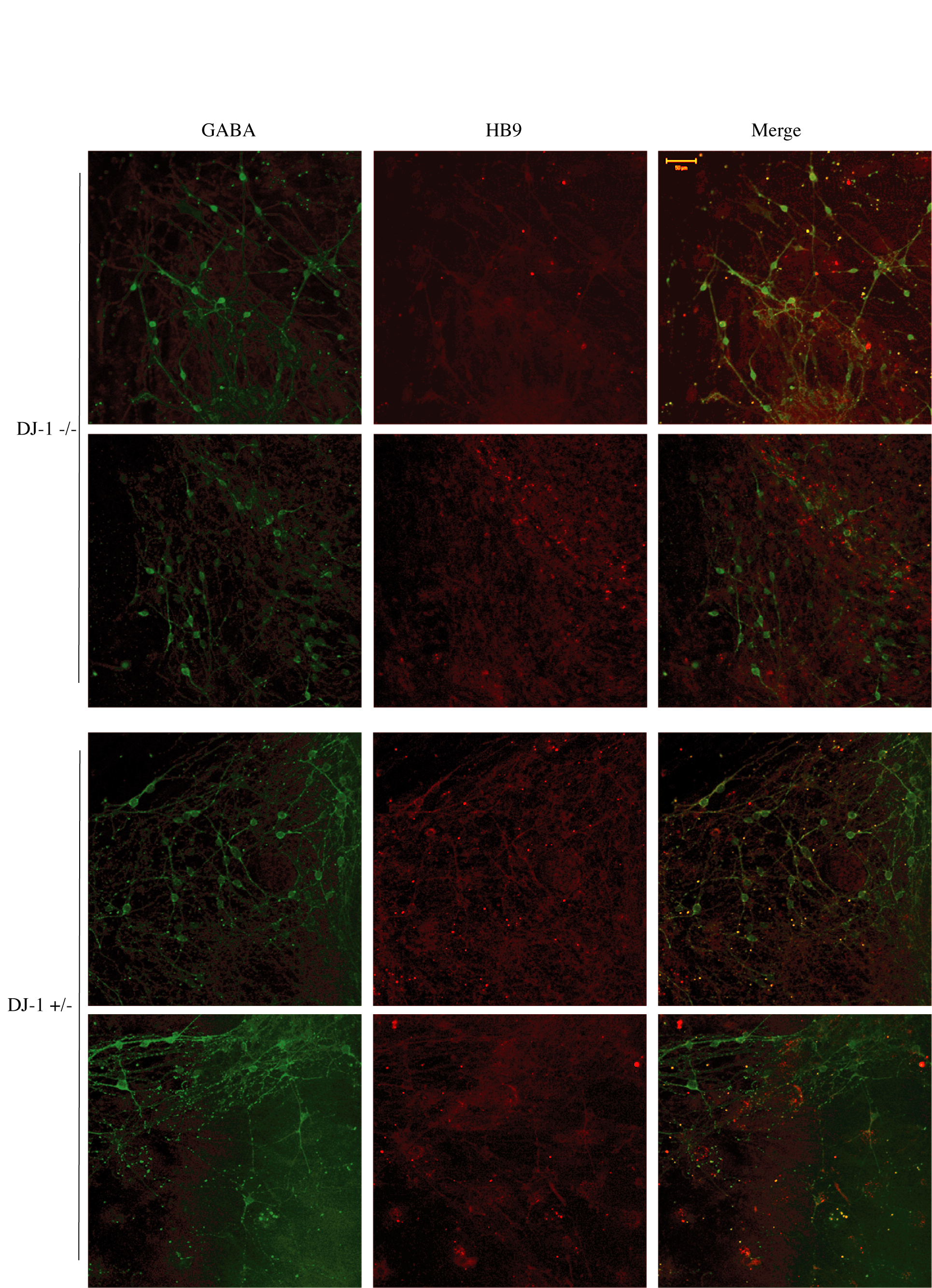

Supplement: Figure S3 — Both cell cultures were differentiated by SDIA for 18 DIV. Cells were fixed with 4% paraformaldehyde and stained with mouse monoclonal antibodies against HB9 (gift from T. Jessell, dilution 1:50) and rabbit polyclonal antibodies against GABA (Sigma, dilution 1:1000) as in Figure 5. Scale bar, 50 μM. (5.5 MB TIF). [file pbio.0020327.sg003.tif]
